# Feasibility and Acceptability of a Student-Led Lifestyle (Diet and Exercise) Intervention Within a Residential Rehabilitation Setting for People With Severe Mental Illness, GO HEART (Group Occupation, Health, Exercise And Rehabilitation Treatment)

**DOI:** 10.3389/fpsyt.2020.00319

**Published:** 2020-04-28

**Authors:** Nicole Korman, Harley Fox, Tina Skinner, Cassandra Dodd, Shuichi Suetani, Justin Chapman, Stephen Parker, Frances Dark, Cheryl Collins, Simon Rosenbaum, Dan Siskind

**Affiliations:** ^1^Addiction and Mental Health Services, Metro South Health Services, Brisbane, QLD, Australia; ^2^School of Medicine, University of Queensland, Brisbane, QLD, Australia; ^3^School of Exercise and Nutrition Sciences, Queensland University of Technology, Brisbane, QLD, Australia; ^4^Queensland Institute of Medical Research, Mental Health and Complex Disorders, Brisbane, QLD, Australia; ^5^School of Psychiatry, Faculty of Medicine, UNSW, Sydney, NSW, Australia

**Keywords:** severe mental illness, schizophrenia, exercise, diet, rehabilitation, lifestyle intervention, physical activity, student placement

## Abstract

**Purpose:**

People with severe mental illness (SMI) experience poor physical health and premature mortality, contributed significantly by modifiable lifestyle risk factors such as poor nutrition, low cardiorespiratory fitness, and physical inactivity. Lifestyle interventions can reduce cardiometabolic risk and confer a range of other positive mental and physical health benefits. We assessed the feasibility, acceptability, safety, and preliminary effectiveness of a lifestyle (combined dietary and exercise) intervention lead by senior exercise and dietetics students in a residential mental health rehabilitation setting.

**Design:**

Single arm, prospective study evaluating outcomes pre and post a 10-week dietary and exercise intervention.

**Method:**

People with SMI from three residential rehabilitation units participated in a mixed aerobic and resistance training exercise intervention three times per week that was combined with a dietary intervention (six individual and group sessions). Primary outcome considerations were feasibility (recruitment, retention, and participation rates), acceptability, and adverse events. Secondary outcomes were preliminary effectiveness; (functional exercise capacity, volume of exercise, and metabolic markers), psychiatric symptoms, quality of life, and attitudes to exercise.

**Results:**

Forty-two participants were recruited (92% primary diagnosis of schizophrenia). Intervention feasibility was supported by high levels of recruitment (68%), retention (77%), and participation (70% exercise, 65% diet sessions); and the absence of serious adverse events. Significant improvements in functional exercise capacity, volume of exercise, general psychiatric symptoms, and negative psychotic symptoms occurred. Anthropometric and metabolic blood markers did not change. While the intervention was acceptable to participants, motivation for and perceived value of exercise reduced over 10 weeks.

**Conclusions:**

A brief pragmatic student-led lifestyle intervention integrated into usual mental health care was feasible, acceptable, safe, and scalable across two additional mental health residential rehabilitation sites, and resulted in physical and mental health improvements. Increased frequency of dietary sessions and length of dietary intervention may improve metabolic outcomes in the future. People with SMI living in residential rehabilitation units should have access to lifestyle programs to address modifiable lifestyle risk factors. While this brief intervention was feasible and acceptable, this study highlights some of the challenges associated with maintaining motivation for healthy lifestyles for people with SMI. Longer term investigation of real-world lifestyle interventions is warranted, together with additional interventions that may support people with SMI to sustain motivation to address lifestyle factors.

**Clinical Trial Registration:**

The trial was registered with the Australian New Zealand Clinical Trials Registry (ANZCTR), Unique Identifier: ACTRN 12618000478213, http://www.anzctr.org.au Universal trial number (UTN)—U1111-1211-4009.

## Introduction

People with severe mental illness (SMI) have poor physical health and experience premature mortality of approximately 15–20 years compared to the general population (1); up to 85% of this prematurely mortality has been attributed to physical illnesses such as cardiometabolic disease (CMD), and cancer (2). Modifiable risk factors such as physical inactivity, poor nutrition, and low cardiorespiratory fitness play a significant contribution to this increased risk (3, 4). While the poor physical health of people with SMI has gained increasing international recognition (5), there is evidence that physical health disparities persist and the mortality gap for people with SMI may actually be widening (1). Addressing modifiable risk factors and improving the physical health of people with SMI should be a primary concern in a mental health setting (5).

Several meta-analyses of lifestyle (diet and exercise) interventions for people with SMI have demonstrated improvements in metabolic risk factors, such as waist circumference, body mass index (BMI), fasting blood glucose, triglycerides, and blood pressure (6–8). A recent meta-review found lifestyle interventions were comparable to pharmacotherapy for cardiometabolic risk reduction (9). However, the majority of lifestyle interventions have been conducted in outpatient community settings, or with early psychosis populations (10).

One setting in which few lifestyle interventions have been conducted are residential rehabilitation and supported housing units for people with SMI. People accessing these services typically experience significant burden from negative and cognitive symptoms, and have lower functioning than people living in community settings (11, 12). In Australia, Community Care Units (CCU’s) are a type of residential rehabilitation which provide independent housing in the community, with access to 24 h on-site multidisciplinary mental health staff, but do not provide food or meals.

Previous lifestyle studies conducted within supported housing, residential rehabilitation, or long stay inpatient units have broadly employed two different methods; either *via* evaluating the effect of the addition of combined structured nutrition and exercise interventions provided directly to residents with SMI (13, 14), or by more broadly focusing on attempts to utilize existing resources to improve the environment and culture of facilities, *via* policy change and by upskilling mental health staff to promote healthy behaviors for residents of the facilities (15–17). Both types of studies have found various improvements in components of the metabolic syndrome [weight, BMI, waist circumference, blood pressure, high density lipoprotein (HDL) cholesterol], (13–17) and objective physical activity (15). In an Australian context, to date, no combined diet and exercise interventions have been formally evaluated within a residential rehabilitation setting.

Our team conducted a small (n = 10) exercise physiology student-led pilot supervised exercise intervention (three sessions per week of supervised exercise) in 2016 in a single residential rehabilitation unit, Coorparoo CCU (18). We found the intervention to be feasible and improved functional exercise capacity and total exercise volume/week. However, no changes in metabolic risk factors were observed. We postulated that this may be due to the poor diet quality of the participants (19), and the addition of a dietary intervention may be beneficial.

In this follow-up study, we expanded on the pilot study by including (i) the dietary intervention (six individual and group sessions), and (ii) two additional CCU sites. Research from large scale clinical trials are often significantly resourced compared to usual clinical settings, with interventions typically led by external research teams, which limits generalizability to usual care settings (20, 21). There are increasing calls for the research agenda of lifestyle interventions to include studies that report outcomes from pragmatic and real-world settings (22, 23). Hence, in the current study, we implemented and evaluated the feasibility of a 10-week, exercise physiology and dietetics student-led, pragmatic lifestyle intervention integrated within the usual mental health care setting of three CCU’s.

Based on the results of our pilot exercise study, we hypothesize that the combined dietary and exercise lifestyle intervention will be feasible and acceptable increase fitness (measured *via* functional exercise capacity) and the amount of exercise of participants. Further, we hypothesize the addition of a dietary component to the intervention will decrease metabolic risk factors of participants with SMI located within CCU’s.

## Materials and Methods

### Study Design

This study is a single arm, prospective trial. The intervention consisted of a 10-week lifestyle intervention conducted on-site at three different CCU’s. This study was approved by the Metro South Human Research Ethics Committee in March 2016 (HREC 16/QPAH/042).

### Participants

Participants were residents of three CCU’s within the Metro South Addiction and Health Services in Brisbane, Australia. The residents were initially assessed by the CCU consultant psychiatrist, using the Diagnostic Statistical Manual of Mental Disorders, Fifth Edition (DSM-5) and admitted to a CCU with a diagnosis of SMI (schizophrenia, schizoaffective disorder, bipolar disorder, or chronic major depression) for psychosocial rehabilitation. Admission criteria for CCU’s stipulate that residents with a history of co-morbid substance abuse are able to access residential services provided they do not use substances on site or access rehabilitation services while intoxicated and are engaged in an active process of seeking help to manage their substance abuse disorder from CCU staff or other agencies. The maximum number of residents living in a CCU at any one time is 20, with an expected length of stay of approximately 12 months.

Participants were recruited to the study *via* verbal invitation from research staff located at each CCU and referral by staff members. Recruitment occurred over a 3-week period prior to the intervention. Inclusion criteria for the study were: (i) a current resident of the CCU (ii) able to provide informed consent, and (iii) aged between 18–65 years. Residents were excluded if they were: (i) currently engaging in exercise of ≥150 min/week of low-to-moderate intensity or ≥75 min/week of vigorous intensity exercise, (ii) considered high risk for aggression or suicide by the treating psychiatrist, (iii) assessed to have an acutely unstable mental state by the treating psychiatrist, (iv) pregnant, or (v) classified as high risk on the Adult Pre-Exercise Screening System (APSS), remove a space after (APSS) (24) and not granted medical clearance for exercise by a general practitioner (GP).

Residents with a history of substance abuse were not excluded from access to the lifestyle intervention provided they were able to agree to CCU admission criteria regarding substance abuse.

Participants were provided with a participant information sheet and written informed consent was obtained.

### Exercise Intervention

Three, 1 h group exercise sessions per week were offered, (45-min session with a 5-min warm up and a 10-min cool down) involving circuit training with a combination of aerobic and strength exercises in the shared courtyard of each CCU for 10 consecutive weeks.

Individuals who missed a session or preferred to exercise alone were offered individual sessions. Further details of the exercise intervention are published elsewhere (25).

Drawing on earlier research, the intervention included several evidence-based motivational strategies to increase participants’ adherence to the program (26). These strategies included validation and encouragement of participants, goal setting, individualization of the program and assistance to identify and problem solve barriers for exercise engagement.

Additionally, exercise circuit stations were designed such that they could be modified (regressed/progressed) to suit individual participant capacity and preference which is an important factor in the design of exercise interventions for people with SMI (26, 27).

The sessions were led by two fourth (final) year exercise physiology (EP) students at each CCU undertaking a 12-week practicum placement, under the supervision of university lecturers and a part time accredited exercise physiologist (AEP).

### Dietary Intervention

Final year masters of dietetics (MDiet) students attended CCU’s fortnightly on rotation from their university, and collaborated with mental health staff to offer the dietary intervention on-site at each of the CCU’s setting. The dietary intervention ran concurrently with the 10-week exercise intervention, with an individual dietary assessment and a healthy cooking/eating group occurring on the same day, once per fortnight, on six occasions. While food was provided for the purpose of the cooking group, residents of CCU’s are supported to shop and cook independently, and no food is provided on site for other meals.

There were three components of the diet intervention:

1. Group education on basic healthy nutrition on the following structured topics: portion size, recommended daily intake, daily energy requirements, benefits of fiber and how to increase daily consumption, label reading, healthy snack and breakfast preparation, incorporation of more vegetables, food storage, and weight loss principles.
2. Cooking skills demonstration where participants practiced basic cooking skills under the supervision of the MDiet students followed by a shared eating experience. Participants were provided with a copy of the recipe broken down into simple steps for future use.
3. On the same day as the cooking group. MDiet students offered individualized dietary assessment and counseling session based on participants’ goals.

Any participants discharged from the CCU prior to the study completion were invited to continue attending both the dietary and exercise sessions at the CCU’s.

The student placements were an opportunity for mutual collaboration and capacity building for both EP students, Mdiet students, and mental health multidisciplinary staff regarding physical activity, nutrition, and mental health concepts (28).

### Outcomes

#### Primary Outcomes: Feasibility, Safety, and Acceptability

Feasibility was determined through procedural statistics (recruitment, retention, participation rates). Previous studies were used to determine thresholds for indicative feasibility *a priori*.

1. Retention: Study feasibility was determined if >70% of the sample complete the study. Previous feasible lifestyle interventions had retention rates of 73%–78% over the first 6 months (29–31).
2. Recruitment: We considered the intervention feasible if the majority of CCU residents (>60%) were recruited. Recruitment is based on the percentage of participants who consent to the intervention as a proportion of residents residing in a CCU during the recruitment phase 3 weeks prior to study onset.
3. Participation rates: The intervention was considered feasible if participants completed ≥65% of the exercise and diet interventions. Fitness improvements have been reported in rehabilitation studies in which participants completed ≥66% of 3*/week classes (32) and weight loss in participants who participated in ≥ 66% of group and individual diet interventions over the first 6 months (30).

Safety was reported as the number of adverse events (new injuries, exacerbation of pre-existing conditions), experienced during the intervention as a proportion of the total number of participants recruited into the trial.

Acceptability of the exercise (questionnaire adapted from previous exercise research in this population), (33) and dietary components of the program were assessed *via* questionnaires on a 5-point Likert Scale (strongly disagree, disagree, neutral, agree, strongly disagree). Responses were collapsed into three categories: disagree/strongly disagree, neutral, and agree/strongly agree.

#### Secondary Outcomes (Preliminary Effectiveness)

1. Functional exercise capacity; a submaximal proxy measure of fitness (34), was measured *via* the distance walked during the 6 Minute Walk Test (6MWT). The 6MWT was performed according to the ATS guidelines (35)
2. Total psychiatric symptoms was measured *via* the Brief Psychiatric Rating Scale (BPRS), (36), a semi-structured interview.
3. Negative symptoms was measured *via* the Scale for the Assessment of Negative Symptoms (SANS), (37).
4. Physical activity levels and sedentary behavior was measured using the Simple Physical Activity Questionnaire (SIMPAQ). The SIMPAQ is a researcher-administered self-report questionnaire assessing time (average minutes and hours) spent in bed, sitting or lying down, napping during the day, walking, structured exercise, and incidental activities completed in the previous week (38).
5. Fasting blood lipids: (total cholesterol, HDL, low-density lipoprotein, and triglycerides) and blood glucose. Venous blood was collected from a trained phlebotomist from a community collection center and analyzed using standard clinical laboratory procedures
6. Anthropometrics: BMI, waist circumference, height (meters), and body weight (kg) were measured on an electronic scale and wall-mounted stadiometer, respectively, for the calculation of BMI, (weight/height^2^). Waist circumference was measured using a retractable anthropometry tape (39)
7. Quality of life: was assessed using the AQol-8D, a reliable and valid instrument, which is particularly suitable when psychosocial elements of health are of importance. It involves questions assessing eight dimensions of mental and physical health; three of the dimensions (independent living, pain, senses) make up a physical super-dimension; the other five (mental health, happiness, coping, relationships, and self-worth) a mental super-dimension. Each dimension has high reliability (Cronbach’s α=0.61–0.96), and a demonstrated ability to distinguish general population scores from mental health patients. Dimension scores are placed scale of 0–1, where 1 is optimal health (40).
8. Participants’ motivations toward exercise was measured by the Behavioral Exercise Regulations Questionnaire (BREQ-2), (41). The BREQ-2 comprises 19 items to assess six domains of motivation; amotivation external, introjected, identified, integrated and intrinsic behavioral regulations (42). Responses to items are scored on a 5-point scale ranging from 0 = “not true for me” to 4 = “very true for me, with an average score created for each of the six domains, with higher scores indicating higher ratings in each of these domains.

### Statistical Analysis

Results were analyzed using Statistical Package for the Social Sciences (SPSS, version 26, IBM). Normality of the resulting model residuals were assessed using the Shapiro-Wilk test and *via* inspection of histogram and quintile-quintile plots. Continuous data was analyzed pre- and post-intervention by paired t-test, or where data were not normally distributed, a Wilcoxon signed rank test. Categorical data was analyzed by chi-squared test. All tests were two-tailed with an alpha of 0.05 applied as the criterion for statistical significance. The Bonferroni correction was conducted to consider significance after multiple analyses performed on the same data. This was achieved by dividing the per analysis rate (*α*_PC_ = 0.05) by the number of statistical analyses performed for all outcome measures. Given 25 statistical tests were performed, individual outcomes remained significant after Bonferroni corrections if *P* ≤ 002, which was indicated in the respective tables with a superscript ^a^. Effect sizes for parametric data were calculated using the Cohen’s d statistic (cut points; low = < 0.4, medium = 0.4–0.6, large = > 0.6) and nonparametric data (z/√n), (cut points; small 0.1, medium 0.3, large 0.5).

## Results

### Baseline Demographics

The primary SMI diagnosis of participants in this study was schizophrenia (93%), with a median of with a median of 7.5 years of illness [inter-quartile range (IQR) = 2.9–10.0 years]. Of the 42 participants recruited to this study, 26 (62%) were taking clozapine or olanzapine, atypical antipsychotic medications with the highest propensity for weight gain, with an average of 3.6 (SD = 2.1) medications per participant.

There were no significant differences between participants who completed or withdrew from the intervention in terms of age, total number of medications, duration of illness, sex, employment, education, diagnosis, smoking status, chronic disease, or antipsychotic medications (**Table 1**). However, participants with a diagnosis of substance abuse were more likely to be in the withdrawal group (p=0.002). Further, there was a significant difference between those who completed follow up assessments and those who did not in total time in rehabilitation, (completers; median 4.5 months, IQR 1.0–9.0), participants who withdrew, median = 1.0 month, IQR 0.5–2.0; p=0.014).

**Table 1:** Participant Characteristics.

	Total	Completers	Withdrawals	t(df) or z	p value
	(n= 42)	(n=32)	(n=10)		
**Age: years**	30 (9)	31 (10)	29 (8)	t(40)=−0.37	0.715
**Sex: n (%)**					
Males:	33 (78)	27 (84)	6(60)		0.181^a^
**Employed, n (%)**	1 (2)	1 (3)	0 (0)		
**Education**: **n (%)**					
High school	27 (64)	18 (56)	9 (90)		0.061^a^
Tertiary	16 (38)	15 (45)	1 (11)		
**Primary diagnosis: n (%)**					
Schizophrenia/schizoaffective	39 (93)	30 (92)	9 (90)		
Bipolar affective disorder	1 (2)	1 (3)	0 (0)		
Major depressive disorder	2 (4)	2 (6)	0 (0)		
Substance abuse disorder	1 (2)	0 (0)	1 (11)		
**Substance abuse, n (%)**	9 (21)	3 (9)	6 (60)		0.002^a^*
**Duration illness: years ^NP^**	7.5 (2.9–10.0)	8.5 (2.1–11.5)	4.0 (3.0–5.5)	z= −1.08	0.286
**Length of time in rehabilitation: months ^NP^**	2.0 (1.0–8.2)	4.5 (1.0–9.0)	1.0 (0.5–2.0)	z= −2.45	0.014*
**Total no. medications, n(%)**	3.8 (2.1)	3.7 (2.0)	3.6 (2.6)		0.892
**Antipsychotic medications: n (%)**					
Clozapine or olanzapine	26 (62)	20 (61)	6 (60)		1.00^a^
**Chronic disease: n (%)**					
HTN:	2 (4.8)	2 (6)	0		
DM:	0 (0)	0	0		
**Metabolic syndrome: n (%)**	9 (21)	8 (24)	1 (11)		0.653^a^
**Smoker: n (%)**	20 (48)	13 (41)	7 (70)		0.152^a^

All measures are presented as mean and standard deviation unless stated otherwise.

NP, Non-parametric: median and interquartile range; n, number of participants; HTN, hypertension; DM, diabetes mellitus.

*p <0.05 = significant, ^a^Fisher’s exact test—when one or more cell has a cell count frequency of < 5.

### Feasibility

**Figure 1:**
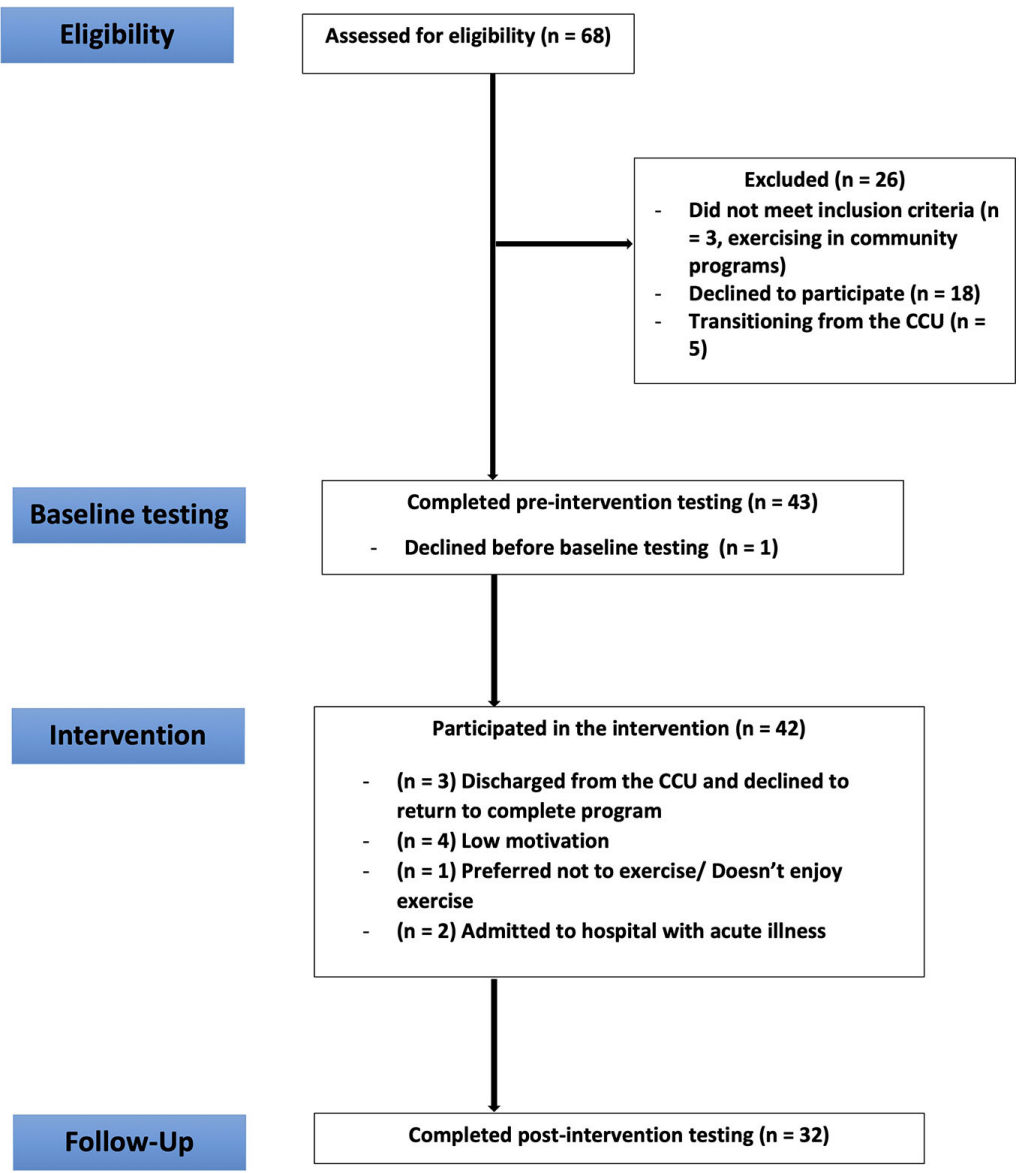
CONSORT diagram.

Of the 68 eligible residents, 42 participants were recruited and completed baseline measures (recruitment rate = 61.8%). Of the 42 recruited participants, 32 completed post-intervention measures and 10 withdrew (retention rate of 77%). Reasons for withdrawal are provided in **Figure 1**.

Participation rate for the 30 available exercise sessions was 70% (of those completing the intervention), (median = 21 sessions, IQR 13.5–30.0). Attendance to the six fortnightly individual and group dietary session was a median of 4 (IQR 3–4).

### Acceptability

Exercise acceptability questionnaires were completed by 50% (n=21) of the sample, (65% of completers), and 62% (n=26) completed the diet acceptability questionnaires, (81% of completers) See **Supplementary Tables 1****–****4**.

Of participants who completed the acceptability questionnaires, the majority expressed satisfaction (86%), enjoyment (90%), desire to continue exercising after the study (71%), and enjoyed the group setting (86%). More participants enjoyed resistance components such as stretch bands (71%), hand weights (76%), and the medicine ball (67%) as compared to cardio elements such as treadmill (47%) or seated bicycle (38%) and more than half preferred more sports to be included. Over half of participants reported they would be confident to complete this exercise on their own and when they were having a bad day (57%).

Of the participants who completed the diet acceptability questionnaire, the majority (88%) would recommend the program to others and noticed improvements in themselves (81%) following the healthy eating program. Eighty-five percent of the participants reported improvement in nutritional knowledge, and 92% of them were willing to continue to use their new skills post-intervention. The dietary intervention was sufficiently challenging for most participants (70%).

### Safety

There were no new injuries or adverse events that resulted in intervention withdrawal. Of the 32 completers, three (9.4%) experienced an exacerbation of pre-existing musculoskeletal issues that required individual modifications to the prescribed exercises without the need for medical intervention. Two participants had an increase in knee pain and one experienced exacerbation of back pain. Modifications were made to avoid aggravating exercises.

### Preliminary Effectiveness

There was a significant increase in functional exercise capacity as measured by 6MWT, mean difference = 56.8 m (95% CI 40–73.66) p< 0.001, large effect size (Cohen’s d = 0.7). Total minutes of exercise increased by 110 minutes per week, post intervention, z=2.24 (p=0.025), medium effect size (r= 0.39) See **Table 2**.

**Table 2:** Pre- and Post-Intervention Measures.

	Baseline	Post	Mean change (95% CI)	t(df) or z	p value
	(n=32)	(n=32)			
**Exercise capacity:**					
6MWT, meters:	476.63 (83.53)	533.47 (88.61)	56.84 (49, 73.6)	t(31) =6.89	<0.001*^b^
**Blood pressure: mmHg**					
Systolic:	121.4 (9.6)	119.7 (8.1)	−1.2(−4.4, 1.9)	t(31)=−0.79	0.435
Diastolic:	79.6 (7.4)	76.4 (7.9)	−2.9 (0.2–5.9)	t(32)=−1.93	0.062
**Anthropometry:**					
BMI: kg/m^2^	28.5 (6.8)	28.7 (7.1)	0.4 (−0.2, 1)	t(32)=1.33	0.192
Waist: cm	100.7 (16.1)	98.4 (18.6)	−0.9 (−0.29, 1.2)	t(31)=−0.84	0.405
**Fasting bloods: mmol/L**					
Blood glucose:	5.3 (0.6)	5.1 (0.5)	−0.05 (−0.2, 0.09)	t(27)=−0.73	0.472
TC: **^NP^**	4.9 (4.4–5.8)	5.2 (4.3–5.8)		z=0.92	0.360
HDL:	1.1 (0.2)	1.29 (0.8)	−0.21 (−0.5–0.1)	t(29)=−1.41	0.170
LDL: **^NP^**	3.0 (2.5–3.7)	3.0 (2.4–3.8)		z=0.64	0.524
Trig: **^NP^**	1.4 (1.0–2.1)	1.5 (1.1–2.1)		z=0.83	0.404
**SIMPAQ data: ^NP^**					
Sedentary: h/day	5.0 (4.0–8.0)	4.0 (3.0–6.5)		z=−0.97	0.332
Mod−Vig: min/week	75 (0–188)	120 (45–255)		z=1.93	0.053
Total Ex volume: min/week	250 (117–417)	360 (208–548)		z=2.24	0.025*
**Psychiatric symptoms:**					
BPRS:	37.7 (11.2)	32.1 (7.4)	4.9 (0.7–9.2)	t(26)=2.42	p=0.023*
**SANS: ^NP^**					
Total:	42 (31–58)	27 (17–38)		z=−4.2	p= < 0.001*^b^
Affect:	17 (10–21)	10 (4–14)		z=−3.01	p=0.003*
Alogia:	2 (0–8)	0 (0–4)		z=−2.50	p=0.012*
Functioning:	7 (4–11)	5 (1–6)		z=−3.17	p=0.002*^b^
Social:	15 (10–18)	12 (8–15)		z=−1.9	p=0.062
Cognitive:	4 (0–7)	1 (0–3)		z=−2.18	p=0.029*
**BREQ: ^NP^**					
Amotivation:	0 (0–.25)	.5 (0–.75)		z=2.66	p=0.008*
External regulation:	.5 (0–2)	.75 (0–1.87)		z=1.29	p=0.196
Introjected Regulation:	1 (0–2.47)	1 (.50–2.17)		z=0.29	p=0.769
Identified Regulation:	3 (2.5–3.5)	2.75 (1.62–3.12)		z=−2.0	p=0.041*
Intrinsic regulation:	3 (2–4)	2.5 (1.87–3.12)		z=−1.4	p=0.146

All measures are presented as mean and standard deviation unless stated otherwise. *p< 0.05 (significant).

^b^Bonferroni correction (≤0.002); CI, confidence interval, NP, non-parametric data presented as median and interquartile range; BMI, body mass index; HTN, hypertension; T2DM, type 2 diabetes mellitus; TC, total cholesterol; HDL, high density lipoprotein; LDL, low density lipoprotein; Trig, triglycerides; n, number of participants; WHR, waist hip ratio; Mod-Vig, moderate to vigorous; Ex, exercise; 6MWT, 6 Minute Walk Test; SIMPAQ, Simple Physical Activity Questionnaire; BPRS, Brief Psychiatric Rating Scale; SANS, Scale for the Assessment of Negative Symptoms.

All measures of psychiatric symptoms reduced following the intervention. Total symptoms reduced by 4.9 points (95% CI 0.7–9.2; p = 0.023), medium effect size (Cohen’s d = 0.6). With respect to negative symptoms, each category of the SANS was reduced with a significant total mean reduction of 15.4 points (95% CI 10.5–20.2; p< 0.001) and large effect size (Cohen’s d = 0.9).

There was a change in the physical SuperDimension of the AQOL-8D, (combined physical health domain: reduced pain, increase in senses; hearing/vision/communication, increase in functioning in independent living skills, mean difference −0.04 (SD 0.12), p = 0.038, small effect (Cohen’s d =0.33), however no change in the psychological SuperDimension or any other individual domain was observed, see **Supplementary Table 5**.

With respect to attitudes to exercise, there was an increase in amotivation (lack of intention to begin exercise) following the intervention (“I can’t see why I would bother exercising, I don’t see the point”), z=2.66, p=0.008, medium effect (r= 0.47), and a reduction in identified regulation (“I value the benefits of exercise, it’s important to exercised regularly”), z= −2.0, p=0.041, medium effect (r= 0.35). There were no other significant differences for any other domain of the BREQ-2.

There were no significant differences from pre- to post-intervention in any of the other outcome measures (sedentary behavior, anthropometric measures, or markers of metabolic health).

## Discussion

Implementation of a brief student-led lifestyle (combined dietary and exercise) intervention integrated into usual mental health care within three residential rehabilitation settings was feasible, safe, and acceptable with no significant adverse events resulting in drop out. We found increases in functional exercise capacity, total exercise volume, and a decrease in negative and total psychiatric symptoms, confirming and extending our 2018 pilot exercise findings across two additional rehabilitation settings. However, addition of the dietary intervention did not improve markers of metabolic health or anthropometric measures.

The recruitment rate of the combined diet and exercise lifestyle intervention (68.0%) was similar to previously reported lifestyle interventions conducted within both outpatient (30, 43) and residential rehabilitation settings (13, 14), (63–70%), indicating the large majority of people accessing rehabilitation facilities may be interested in participating in lifestyle interventions. This is encouraging given that rehabilitation facilities typically have a holistic focus and are often resourced with shared cooking facilities making these settings opportune for implementation of lifestyle interventions (43).

Our retention rates (77%) were high and comparable with other lifestyle intervention studies in SMI populations (73–84%) (29, 30, 43, 44). Features of our program that may have promoted participant retention included support by mental health staff to participate, provision of the intervention on site, and integration into usual mental health care (26).

Participation rate for those completing the exercise intervention was 70%, and was broadly comparable to the 72% reported in a meta-analysis of exercise interventions for people with SMI (45). Of the 32 completers, one third (9) of participants attended 100% of the sessions and were observed to make friendships with other participants during partnering and teamwork involved with circuit sessions, which likely facilitated autonomous motivation to exercise (46). As was highlighted in previous pilot work, (25) an advantage of the student placement design was that EP students had the flexibility to offer both group and individual make-up sessions which increased participation rates. Broadening the exercise program to include less structured exercise formats and greater variety (45), i.e. more sports and use of green spaces (47), in keeping with participants’ preferences, may increase participation further.

For those who completed exercise questionnaires, there was overall acceptance and enjoyment of the exercise program. Confidence to exercise alone and when having a bad day was particularly encouraging as exercise could be included as a coping strategy for symptom exacerbations in the setting of treatment resistance, commonly encountered by residents in residential facilities (12). We found resistance training elements were popular; future exercise interventions could focus on expanding the provision of resistance training, particularly given emerging evidence for this type of training for depressive symptoms, quality of life, and symptoms of psychosis (48–50).

While the brief intervention was acceptable to participants, the most common reason for withdrawal was a lack of motivation to exercise. Additionally, for those completing the intervention we found an increase in amotivation and decrease in the value of exercise on the BREQ-2 questionnaire over ten weeks. Together, these findings confirm the challenges people with SMI can face regarding motivation to adapt and sustain a healthy lifestyle (27), which raises two points. Firstly, while participation was high over ten weeks, amotivation was already increasing which may impact longer term engagement. The vast majority of lifestyle intervention evidence has been reported from studies under 6 months (10), hence longer pragmatic interventions need to be evaluated, as these findings may have a significant impact on lasting health benefits for people with SMI. Secondly, these findings highlight the importance of addressing autonomous motivation when delivering lifestyle interventions (51). While we employed motivational strategies, students leading the lifestyle intervention were not fully qualified; and interventions delivered by qualified practitioners may improve adherence and motivation (52). In future, studies could also investigate the use of objective physical activity measurement devices (i.e. pedometers or accelerometers) and mobile phone apps to target autonomous motivation to address lifestyle behaviors (53–55). Additionally, in future, utilizing principles of co-design, by consulting service users regarding optimal development and implementation of exercise interventions, may increase autonomous motivation to engage (23, 56, 57).

There was broad level of acceptance of the dietetic program by those completing acceptability questionnaires: the majority felt that they had improved their eating habits and would continue to use healthy cooking skills independently, however this was not assessed objectively.

While no adverse events resulted in participant withdrawal, several participants experienced exacerbations of pre-existing injuries that required program modifications. This highlights the importance of exercise professionals prescribing and delivering exercise programs for people with SMI, who are known to experience higher levels of pain and physical comorbidity than the general population (26, 58, 59). Nonetheless, all participants with pre-existing injuries were able to complete a modified program, in keeping with several recent studies suggesting that exercise is safe in people with SMI (22, 51).

Consistent with our hypothesis, and pilot study findings, we found a significant increase in functional exercise capacity (26) of 57 m—these findings are in keeping with the literature regarding exercise interventions and people with SMI (26, 60). Increases of greater than 50 m (achieved by over half of those completing our program) have been associated with clinically significant improvements in walking capacity in people with cardiovascular disease (29, 61) and at improving self-perceived fitness levels in other disease populations (62). Our results were higher than several outpatient lifestyle interventions (increases of 7–34 m), (29, 43, 44), however the average BMI of participants in these studies (BMI 34–37) was higher than ours (BMI 28.5), where obesity is known to cause impairments in functional exercise capacity (63). Our functional exercise capacity increases may also have been higher than other lifestyle studies in outpatients and residential rehabilitation studies due to the greater frequency of sessions we offered (three supervised sessions per week versus one), (13, 29, 44). Engagement in three or more sessions per week has been associated with greater fitness improvements for people with SMI (60), which may have relevance when planning exercise interventions in a residential rehabilitation setting.

There were no significant anthropometric or metabolic changes following our 10-week study. Previous lifestyle interventions in both residential and outpatient rehabilitation settings have demonstrated various reductions in anthropometric (weight, BMI, waist) and metabolic syndrome components in people with SMI (13, 14, 30, 64). There may be several reasons for this finding.

Firstly, people accessing 24 h residential services can have substantial motivational and cognitive deficits (11, 65) and may require more intensive support to integrate changes into their diet. The number of diet intervention sessions we offered (six same-day cooking groups and individual sessions) may have been insufficient to facilitate behavior change. Previous research in both a supported housing and outpatient rehabilitation setting demonstrated, improvements in metabolic syndrome parameters (HDL, systolic BP, body weight) occurred after substantially more diet interventions than our study employed (35 group sessions and 21 combined individual and group sessions respectively), (13, 30).

Secondly, whereas weight loss tends to peak early after lifestyle interventions in the general population, weight loss may occur more gradually for people with SMI (14, 30, 66), hence our 10-week intervention may have been too short to detect significant change.

Thirdly, CCU’s provide support to residents to cook and shop independently but do not provide food on site. Successful weight loss has been reported in rehabilitation settings where food was provided (14, 30) and hence caloric intake and nutrition quality could be modified more easily than in a CCU where participants had to shop for and cook their own food. Healthy meal delivery to replace meals for some CCU residents who experience ongoing barriers to cooking healthy meals regularly despite attempted engagement in rehabilitation to increase independent cooking skills (ie prominent negative and cognitive symptoms), may be an option to improve metabolic outcomes for some residents in future, which may be preferable to “take-away” and discretionary food, with the caveat that this option may increase dependency on external services and need to be weighed carefully against the priority of rehabilitation services to increase independent functioning.

Finally, our sample included participants with chronic illness (mean 8.5 years) in which more than half (62%) were taking the most metabolically unfavorable medications (clozapine and olanzapine), known to substantially increase body weight *via* hunger and metabolic disturbances (67, 68). Greater anthropometric reductions have been found in samples where olanzapine and clozapine prescriptions were much lower (30) and when dietary interventions have been conducted at antipsychotic initiation, highlighting the importance of early intervention (69).

Of note, our participants increased their fitness while maintaining their weight during the 10-week intervention. This may be a more realistic cardiometabolic disease risk reduction goal for people living with longer term exposure to metabolically unfavorable antipsychotics, and particularly in this population with high levels of treatment resistance (11), where switching to antipsychotics with a more favorable cardio-metabolic profile may not be possible due risk of mental state deterioration.

There was a significant reduction in negative symptoms of schizophrenia and general psychiatric symptoms following the lifestyle intervention, consistent with the literature regarding exercise interventions and people with SMI (45, 70). While we observed improvements in social functioning during exercise participation in a subset of our participants, this was a small, uncontrolled study and improvements in symptoms could have occurred due to other aspects of the rehabilitation treatments available at the CCU. We found the physical health component of quality of life improved, confirming the physical health focus of the intervention, however none of the psychological quality of life domains improved. This may relate to the length of the intervention (14, 71), that the lifestyle intervention was not specifically designed to improve psychological constructs (72), or that weight loss did not occur in our participants (a pathway to improved self-efficacy and quality of life) (73).

This brief student-led diet and exercise intervention was found to be feasible, acceptable, and safe for senior students to lead while on practicum placement and could be replicated across residential rehabilitation facilities with minimal resources. Results of this brief study led to the development of an ongoing relationship with local universities providing senior MDiet and EP students on practicum placement at each of the sites throughout the year, resulting in the provision of student-led diet and exercise service to people living in CCU’s. It is beyond the scope of this study to report cost-effectiveness data, however future studies should address this as it is a key factor in determining successful implementation (23). While senior MDiet, EP students and mental health staff were afforded mutual learning opportunities in this novel design, qualified exercise physiology and dieticians could achieve better adherence and physical and mental health outcomes (52, 69).

This was a small, unblinded, uncontrolled pilot study. Psychiatric symptom scales were most prone to observer bias, and these results should be interpreted with caution and future real-world studies in a residential setting should include a control group. Preliminary effectiveness revealed improvements in a number of mental and physical health outcomes, however when multiple analyses were corrected for, only exercise capacity and negative symptoms remained significant; this was a small feasibility study and larger studies are required to confirm our results.

Additionally, while motivational strategies were employed by students, we did not utilize a specific behavior change theory to inform intervention design, limiting discussion regarding implications for clinical practice in light of the reduction in motivation we found and also limiting potential replication of the design in real world settings. Future lifestyle intervention studies in residential settings should utilize and articulate a clear behavior change model (74). While commonly utilized, physical activity self-report measures are prone to recall bias (75), particularly in this population of people with SMI utilizing residential services who can experience significant cognitive impairment. Future studies using device-based measures, (ie accelerometry), could detect differences in sedentary behavior and physical activity more objectively, and particularly as recent research has reported accelerometry may be the most preferable measure of PA by people with SMI (76). Participants reported improvements in their diet following the intervention, however we did not measure energy consumption, diet quality or macro- and micro-nutrient intake, and this should be an important focus in future dietary interventions. Finally, the lower completion rate of the acceptability questionnaires may not have been representative of the entire sample recruited into the study.

## Conclusion

Implementation of a brief student-led lifestyle intervention within mental health residential rehabilitation facilities for people with SMI is feasible, safe, scalable, and may improve functional exercise capacity, exercise participation, and negative symptoms. This pragmatic study may be relevant to other CCU’s and other residential rehabilitation units. People living in CCU’s may require more intensive dietetic support to positively impact metabolic risk. Longer duration of lifestyle interventions and investigation of additional interventions to support sustaining healthy lifestyles are warranted.

## Data Availability Statement

The datasets generated for this study are available on request to the corresponding author.

## Ethics Statement

The studies involving human participants were reviewed and approved by Metro South Human Research Ethics Committeein March 2016: (HREC 16/QPAH/042). The patients/participants provided their written informed consent to participate in this study.

## Author Contributions

NK wrote the protocol in conjunction with DS. CD, NK, SP, CC, and SS collected data. NK, CD, and HF entered data. NK, HF, and DS performed statistical analyses in conjunction with TS. CD supervised the exercise intervention. NK and HF wrote the first draft of the manuscript. All authors NK, HF, CD, TS, JC, SS, DS, FD, CC, and SR revised the final manuscript.

## Conflict of Interest

The authors declare that the research was conducted in the absence of any commercial or financial relationships that could be construed as a potential conflict of interest.
