# Supplementary material for: Feasibility and Acceptability of a Student-Led Lifestyle (Diet and Exercise) Intervention Within a Residential Rehabilitation Setting for People With Severe Mental Illness, GO HEART (Group Occupation, Health, Exercise And Rehabilitation Treatment)
Source: Front Psychiatry. 2020 Apr 28;11:319. doi: 10.3389/fpsyt.2020.00319 (PMC7198865; doi:10.3389/fpsyt.2020.00319)
Supplement: Supplementary file 1 [file Table_1.pdf]

Supplementary Table 1 - Dietetics Program Acceptability Questionnaire (n=26)	Poor<br>n (%)	Average<br>n (%)	Excellent<br>n (%)
How would you rate our experience attending the UQ dietetics student program?	1 (4)	6 (23)	19 (73)
	**Disagree<br>n (%)**	**Neutral<br>n (%)**	**Agree<br>n (%)**
Was your UQ dietetics student program challenging enough for you?	4 (15)	4 (15)	18 (70)
Do you feel your knowledge of healthy eating has improved?	0 (0)	4 (15)	22 (85)
Would you like to continue with your using these skills after the study?	1 (4)	1 (4)	24 (92)
Would you recommend this UQ dietetics student program for other people?	1 (4)	2 (8)	23 (88)
Have you noticed other improvements in yourself following this healthy eating program?	0 (0)	5 (19)	21 (81)
