# Supplementary material for: Feasibility and Acceptability of a Student-Led Lifestyle (Diet and Exercise) Intervention Within a Residential Rehabilitation Setting for People With Severe Mental Illness, GO HEART (Group Occupation, Health, Exercise And Rehabilitation Treatment)
Source: Front Psychiatry. 2020 Apr 28;11:319. doi: 10.3389/fpsyt.2020.00319 (PMC7198865; doi:10.3389/fpsyt.2020.00319)
Supplement: Supplementary file 2 [file Table_2.pdf]

Supplementary Table 2 - Exercise Program Acceptability Questionnaire (n = 21)	Disagree n (%)	Neutral n (%)	Agree n (%)
I feel satisfied with the exercise program	1 (4)	2 (10)	18 (86)
I would like to continue this program after the study ends	2 (10)	4 (19)	15 (71)
I found the exercise program enjoyable	2 (10)	0 (0)	19 (90)
I found the seated bicycle enjoyable	2 (10)	11 (52)	8 (38)
I found the exercise boring	16 (76)	2 (10)	3 (14)
I would have preferred exercising individually (not in a group)	9 (43)	9 (43)	3 (14)
I looked forward to the exercise sessions	4 (19)	3 (14)	14 (67)
I found the stretching bands enjoyable	3 (14)	3 (14)	15 (71)
I would feel confident doing this kind of exercise without assistance	3 (14)	4 (19)	14 (67)
I found the boxing elements enjoyable	3 (14)	8 (38)	10 (48)
I would feel confident being able to do this kind of exercise when having a bad day	5 (24)	4 (19)	12 (57)
I found the treadmill/walking elements enjoyable	2 (10)	9 (43)	10 (48)
I found the following strength elements enjoyable: hand weights	2 (17)	3 (14)	16 (76)
I found the medicine ball enjoyable	4 (19)	3 (14)	14 (67)
I found the exercise too hard	11 (52)	9 (43)	1 (4)
I found the group setting enjoyable	1 (4)	2 (10)	18 (86)
I would have preferred more sports in the exercise program	3 (14)	6 (29)	12 (57)
I would have liked the exercise program to be more challenging	8 (38)	10 (48)	3 (14)
I found the rope enjoyable	5 (24)	4 (19)	12 (57)
I wish the exercise program was more fun	9 (43)	4 (19)	8 (38)
