# Supplementary material for: Feasibility and Acceptability of a Student-Led Lifestyle (Diet and Exercise) Intervention Within a Residential Rehabilitation Setting for People With Severe Mental Illness, GO HEART (Group Occupation, Health, Exercise And Rehabilitation Treatment)
Source: Front Psychiatry. 2020 Apr 28;11:319. doi: 10.3389/fpsyt.2020.00319 (PMC7198865; doi:10.3389/fpsyt.2020.00319)
Supplement: Supplementary file 3 [file Table_3.pdf]

Table 3 - Dietetics Program Acceptability Questionnaire, complete responses<br>N=26	Very poor	Poor	Neutral	Good	Excellent	
	**1<br>n(%)**	**2<br>n(%)**	**3<br>n(%)**	**4<br>n(%)**	**5<br>n(%)**	
How would you rate our experience attending the UQ dietetics student program?	0	1(4)	6(23)	16(61)	3(11)	N=26
	**Strongly disagree**	**Disagree**	**Neutral**	**Agree**	**Strongly Agree**	
	**1<br>n(%)**	**2<br>n(%)**	**3<br>n(%)**	**4<br>n(%)**	**7<br>n(%)**	
Was your UQ dietetics student program challenging enough for you?	0	4(15)	4(15)	11(42)	7(27)	N=26
Do you feel your knowledge of healthy eating has improved?	0	0	5(19)	14(53)	7(27)	N=26
Would you like to continue with your using these skills after the study?	0	1(4)	1(4)	20(76)	4(15)	N=26
Would you recommend this UQ dietetics student program for other people?	0	1(4)	2(8)	15(58)	8(31)	N=26
Have you noticed other improvements in yourself following this healthy eating program?	0	0	5(19)	14(53)	7(27)	N=26
