# Supplementary material for: Feasibility and Acceptability of a Student-Led Lifestyle (Diet and Exercise) Intervention Within a Residential Rehabilitation Setting for People With Severe Mental Illness, GO HEART (Group Occupation, Health, Exercise And Rehabilitation Treatment)
Source: Front Psychiatry. 2020 Apr 28;11:319. doi: 10.3389/fpsyt.2020.00319 (PMC7198865; doi:10.3389/fpsyt.2020.00319)
Supplement: Supplementary file 4 [file Table_4.pdf]

Supplementary Table 4, Exercise Acceptability Questionnaire, complete responses on likert scale

		Strongly Disagree n(%)	Disagree n(%)	Neither agree nor disagree n(%)	Agree n(%)	Strongly Agree n(%)
a	I feel satisfied with the exercise program		1(5)	2(9)	13(62)	5(24)
b	I would like to continue this program after the study ends		2(9)	4(19)	11(52)	4(19)
c	I found the exercise program enjoyable		2(9)		16(76)	3(14)
d	I found the seated bicycle enjoyable		2(9)	11(52)	8(38)	
e	I found the exercise boring	3(14)	13(62)	2(9)	3(14)	
f	I would have preferred exercising individually (not in a group)		9(43)	9(43)	2(9)	1(5)
g	I looked forward to the exercise sessions		4(19)	3(14)	12(57)	2(9)
h	I found the stretching bands enjoyable		3(14)	3(14)	14(67)	1(5)
i	I would feel confident doing this kind of exercise without assistance	1(5)	2(9)	4(19)	8(38)	
j	I found the boxing elements enjoyable		3(14)	8(38)	9(43)	1(5)
k	I would feel confident being able to do this kind of exercise when having a bad day	1(5)	4(19)	4(19)	12	
m	I found the treadmill/walking elements enjoyable		2(9)	9(43)	8(38)	2(9)
n	I found the following strength elements enjoyable: hand weights		2(9)	3(14)	15(71)	1(5)
o	I found the medicine ball enjoyable		2(9)	3(14)	14(67)	
p	I found the exercise too hard	1(5)	10(48)	9(43)	1(5)	
r	I found the group setting enjoyable		1(5)	2(9)	15(71)	3(14)
s	I would have preferred more sports in the exercise program		3(14)	6(29)	8(38)	4(19)
t	I would have liked the exercise program to be more challenging	1(5)	7(33)	10(48)	3(14)	
u	I found the rope enjoyable	1(5)	4(19)	4(19)	11(52)	1(5)
v	I wish the exercise program was more fun	2(9)	7(33)	4(19)	5(24)	3(14)
