# Supplementary material for: Feasibility and Acceptability of a Student-Led Lifestyle (Diet and Exercise) Intervention Within a Residential Rehabilitation Setting for People With Severe Mental Illness, GO HEART (Group Occupation, Health, Exercise And Rehabilitation Treatment)
Source: Front Psychiatry. 2020 Apr 28;11:319. doi: 10.3389/fpsyt.2020.00319 (PMC7198865; doi:10.3389/fpsyt.2020.00319)
Supplement: Supplementary file 5 [file Table_5.docx]

**Supplementary Table 5, Quality of Life (AQOL) results**

| **Paired Samples Test** | | | | | | | | | |
| --- | --- | --- | --- | --- | --- | --- | --- | --- | --- |
|  | | Paired Differences | | | | | t | df | Sig. (2-tailed) |
|  |  | Mean | Std. Deviation | Std. Error Mean | 95% Confidence Interval of the Difference | |  |  |  |
|  |  |  |  |  | Lower | Upper |  |  |  |
| Pair 1 | AQoL8D Dim1 Independent Living value - vILpost | -.03036 | .16673 | .02995 | -.09151 | .03080 | -1.014 | 30 | .319 |
| Pair 2 | AQoL8D Dim7 Pain value - vPapost | -.03882 | .13169 | .02365 | -.08712 | .00948 | -1.641 | 30 | .111 |
| Pair 3 | AQoL8D Dim8 Senses value - vSpost | -.02321 | .14473 | .02599 | -.07629 | .02988 | -.893 | 30 | .379 |
| Pair 4 | AQoL8D vSuperDimPhysical value - vSuperDimPhysicalpost | -.04939 | .12652 | .02272 | -.09579 | -.00298 | -2.173 | 30 | .038 |
| Pair 5 | AQoL8D Dim3 Mental Health value - vMHpost | -.00039 | .08440 | .01516 | -.03135 | .03057 | -.026 | 30 | .980 |
| Pair 6 | AQoL8D Dim2 Happiness value - vHappost | .00773 | .12571 | .02258 | -.03838 | .05384 | .342 | 30 | .735 |
| Pair 7 | AQoL8D Dim4 Coping value - vCoppost | -.00320 | .16072 | .02887 | -.06215 | .05575 | -.111 | 30 | .913 |
| Pair 8 | AQoL8D Dim5 Relationships value - vRelpost | -.02105 | .13039 | .02342 | -.06888 | .02677 | -.899 | 30 | .376 |
| Pair 9 | AQoL8D Dim6 Self Worth value - vSWpost | -.00858 | .13373 | .02402 | -.05764 | .04047 | -.357 | 30 | .723 |
| Pair 10 | AQoL8D vSuperDimMental value - vSuperDimMentalpost | .00167 | .15503 | .02784 | -.05520 | .05853 | .060 | 30 | .953 |
| Pair 11 | Utility Score for AQoL-8D - AQoL8DUtilitypost | -.02175 | .12416 | .02230 | -.06729 | .02379 | -.975 | 30 | .337 |
